# Qualitative Perspectives of Young-Onset Dementia Care in Australia: A Thematic Analysis From the Joint Solutions Project

**DOI:** 10.1177/14713012251360600

**Published:** 2025-07-12

**Authors:** Clare Beard, Priscilla Tjokrowijoto, Debbie Stange, Nathan M. D’Cunha, Naomi Moylan, Monica Cations, Adrienne Withall, Jade Cartwright, Samantha M. Loi

**Affiliations:** 1 College of Education, Psychology and Social Work, 1065 Flinders University, Adelaide, SA, Australia; 2 Neuropsychiatry Centre, 90134 Royal Melbourne Hospital, Parkville, VIC, Australia; 3 95923 Wesley College, Melbourne, VIC, Australia; 4 Centre for Ageing Research and Translation, 110446 University of Canberra, Bruce, ACT, Australia; 5 95974 Brightwater Care Group, Inglewood, WA, Australia; 6 School of Psychology, Faculty of Science, 7800 University of New South Wales, NSW, Australia; 7 School of Health Sciences, 3925 University of Tasmania, Launceston, TAS, Australia; 8 Department of Psychiatry, 90134 University of Melbourne, Parkville, VIC, Australia

**Keywords:** young-onset dementia, dementia, dementia care pathway

## Abstract

People with young-onset dementia in Australia face significant challenges due to inconsistencies in diagnostic and post-diagnostic care. These challenges stem from a general lack of knowledge of young-onset dementia, limited age-appropriate supports, and inconsistent services that are available for people with young-onset dementia. The lack of awareness, insufficient professional training, and inadequacies in services exacerbate these challenges, and geographic disparities exist. In 2024, the J*oint Solutions Young-onset Dementia* project aimed to explore the care experiences of people with young-onset dementia in Australia, highlighting gaps, barriers, and positive aspects of the care pathway This paper presents the emergent themes derived from the *Joint Solutions* project. Using qualitative methodology, we gathered optional free-text data from a pool of 313 survey participants and feedback from 47 focus group participants, including individuals with young-onset dementia, caregivers, general practitioners, and community service providers. A thematic analysis generated six key recurring themes and patterns underscoring the needs of this population. These themes highlight the necessity to (1) increase *knowledge, information, and education* relating to young-onset dementia; (2) improve services to manage the *psychosocial implications* associated with young-onset dementia; (3) ensure sufficient *requisites* are in place to provide necessary support and preparedness; (4) enhance the *accessibility and availability* of age-relevant services; (5) consider the impact young-onset dementia has on personal *finances*; (6) reduce *bureaucracy* to obtaining support. These findings emphasise the urgent need for a nationally consistent care pathway to address young-onset diagnostic and post-diagnostic care. A comprehensive framework must be developed, offering a diverse range of resources and services tailored to meet the unique needs of individuals diagnosed with young-onset dementia and their families.

## Introduction

Young-onset dementia, defined as the onset of dementia before the age of 65 (Cartwright et al., 2021; Draper & Withall, 2016), has a global prevalence estimated at 119 per 100,000 population aged 30 to 64 years (Hendriks et al., 2021; Loi et al., 2023) and accounts for up to 10% of all dementia (Cations et al., 2022). Causes of young-onset dementia are heterogeneous and include a higher prevalence of rarer types of dementia at a younger life stage (Stamou et al., 2022). However, well-known dementias such as Alzheimer’s disease and vascular and frontotemporal dementia remain common (Loi et al., 2023), and differential diagnoses can be broad (Tsoukra et al., 2022). Young-onset dementia has a higher instance of familial and secondary dementias compared to late-onset dementia (Draper & Withall, 2016), including injuries and substance use (Cations et al., 2021).

Diagnostic services for young-onset dementia are provided through Australia’s Medicare system. This publicly funded universal public healthcare system covers a significant portion of costs for general practitioner visits and consultants, as well as certain medical tests and pharmaceuticals. However, those with private health insurance can instead utilise this to provide greater choice and access. Most post-diagnostic support for young-onset dementia is accessed through the National Disability Insurance Scheme which uses individualised funding for reasonable and necessary support (Cations et al., 2022). For context, the National Disability Insurance Scheme is administered through an overarching agency that holds funds and approves individual support packages for eligible people under age 65. Applicants are individually assessed for need and not every person with young-onset dementia is approved for a funded National Disability Insurance Scheme package. For example, people must be under age 65 at the time of application, regardless of whether they received their diagnosis before age 65. Applicants must present with permanent and significant disability to qualify for access to the National Disability Insurance Scheme. This may exclude those who lack severity of impairment at the time of application. The cognitive nature of young-onset dementia may cause delay or contribute to incomplete submissions for otherwise qualifying applicants, or assessors may not fully understand or recognise the extent of disability that presents with young-onset dementia and reject the application. Prior to the National Disability Insurance Scheme, Dementia Australia, a national peak body, was funded to provide a specialist young-onset dementia key worker program to offer expert information and advice, including post-diagnostic support. However, this program ceased its intake in 2016 with the expectation that this service would transition to the National Disability Insurance Scheme by 2018 (Dementia Australia, 2018).

Previous studies from outside Australia focusing on post-diagnostic aspects of young-onset dementia have investigated behaviour support (Cadwallader et al., 2023) and caregiver distress (Kang et al., 2022), while a systematic review identified age-appropriate post-diagnostic services for young-onset dementia and their effectiveness (Mayrhofer et al., 2018). These studies identified several deficiencies in young-onset dementia care, including access issues, long wait times, a lack of adherence to best-practices for behaviour support, and the need for ongoing psychoeducation. Furthermore, the impact of young-onset dementia may be alleviated by implementing evidence-based, co-designed age-appropriate supports, which may also help delay institutionalised care for people with young-onset dementia.

There remains a gap in the literature of recent in-depth perspectives about young-onset dementia care of all stakeholders who experience young-onset dementia and provide care in Australia. A qualitative study investigated insights of health professionals in Australia around multi-level barriers and facilitators for accurate and timely young-onset dementia diagnosis and found a need for tailored diagnostic care (Burkinshaw et al., 2023). However, this study did not explore aspects of post-diagnostic care and experiences of people with young-onset dementia, caregivers, and those involved in the young-onset dementia care pathway are not yet fully understood. Recognising these experiences will be essential for the evolution of effective young-onset dementia care.

The *Joint Solutions* project was commissioned by the Australian Government Department of Social Services to identify a gold-standard pathway of dementia care for individuals with young-onset dementia in Australia (Loi et al., 2024, 2025). This paper reports on a thematic analysis of the qualitative data gathered from that project, comprising optional free-text questionnaire comments and focus group discussions from people with young-onset dementia, their caregivers, clinicians, and service providers. Free-text responses provide nuanced insights that quantitative data alone cannot capture, enhancing context and meaning (Rich et al., 2013), while focus group discussions systematically gather detailed information through open-ended questions (Weller et al., 2018). Together, they provide a rich data source for understanding the current experiences of people with young-onset dementia and their families in Australia.

## Methodology

### Study Design

We used a critical realist grounded theory approach to analyse qualitative data gathered in the *Joint Solutions* project. This approach blends critical realism (a philosophy of science) with grounded theory (building theories from data) to ask, *“what makes this possible?”* (Looker et al., 2021; Oliver, 2012, p. 380) and help understand what is happening, why and how it happens, including underlying causes and structural conditions. This approach ensured the analysis captured both the intent of commentary and the environmental and systemic factors affecting diagnostic and post-diagnostic care experiences. Ethics approval for the *Joint Solutions* project was obtained from The University of Melbourne Human Research Ethics Committee (Project ID #28541).

### Participants and Data Collection

Participant recruitment for the survey has been previously reported (Loi et al., 2025). Surveys were created using input from a working group within the Australian Young-onset Dementia Special Interest Group network. A total of 313 participants across all states and territories completed the survey, with some participants providing optional free-text commentary at various points. The surveys addressed the complete care continuum, from pre-diagnosis through to palliative care and provided options for the addition of free-text commentary. Different surveys were developed for each target group (clinicians, GPs, service providers, people with young-onset dementia, and caregivers). We distributed the links to surveys across a wide range of clinical, health care, and community-based settings suggested by the Australian Young-onset Dementia Special Interest Group network, known contacts, and from completing a broad-based internet search. We requested that organisations forwarded survey completion requests across their workforce and to other relevant organisations. Survey participation was open from 27 March to 30 June 2024.

Informed consent was obtained prior to participants proceeding with the survey, which included the use of anonymised data in the findings of the Joint Solutions study. Participant survey responses were kept anonymous, and participants were allowed to withdraw from the survey at any time. On completion, participants could optionally enter a draw for $100 gift voucher. This paper emphasises the qualitative free-text comments that participants could choose to provide in the surveys. The quantitative data arising from the surveys is reported separately in Loi et al. (2025).

Focus group participants were recruited from a post-survey expression of interest. Ten focus groups were conducted between 6 June and 8 July 2024, using the Zoom video conferencing platform. Written consent was obtained from each participant ahead of each focus group, which included agreeing to being audio-visually recorded for transcription purposes. From 143 survey respondents who expressed interest in participating, 75 individuals were invited based on various demographics to ensure broad representation, including location, role, and gender. A total of 47 individuals were able to participate due to availability to attend the scheduled dates and times. We aimed to limit focus groups to a maximum of eight participants and less for those with lived experience who may require sufficient time to provide full answers. There were two groups each for people with young-onset dementia and community service providers, and three groups each for caregivers and clinicians. Community service providers are allied health, other professionals, National Disability Insurance Scheme coordinators and other organisations who provide community-based services to people with young-onset dementia. There were two co-facilitators for every focus group, including one who was representative of the relevant stakeholder group. For example, a person with caregiver experience co-facilitated the caregivers focus group. We conducted two facilitator workshops prior to the focus groups. This consolidated planning for timelines and the focus group duration of 1 hour. There was an opportunity for facilitators to discuss roles, the structure and approach used to maximise discussion, flow of questions, and strategies to manage should participants become uncomfortable or distressed. From the 47 focus group participants, at least *n* = 10 came from each stakeholder group (Table 1). Focus group recordings were transcribed and deidentified prior to data analysis. . All focus group participants received a $100 AUD voucher for their time.

**Table 1.** Demographics of Focus Group Participants

	Person with YOD	Caregiver	Clinician	Community service provider
	*n = 10*	*n = 13*	*n = 14*	*n = 10*
Metropolitan	6 (60%)	8 (62%)	10 (71%)	10 (100%)*
Female	6 (60%)	7 (54%)	11 (79%)	8 (80%)
State				
Victoria	1 (10%)	3 (31%)	3 (21%)	2 (20%)
New South Wales	2 (20%)	3 (31%)	3 (21%)	0 (0%)
Queensland	2 (20%)	2 (15%)	1 (7%)	1 (10%)
South Australia	1 (10%)	2 (15%)	2 (14%)	2 (20%)
Northern Territory	1 (10%)	0 (0%)	2 (14%)	0 (0%)
Western Australia	0. (0%)	2 (15%)	1 (7%)	3 (30%)
Tasmania	1 (10%)	0 (0%)	1 (7%)	1 (10%)
Australian Capital Territory	2 (20%)	1 (8%)	1 (7%)	1 (10%)

*Two Community Service Providers extend their service to regional, rural or remote areas.

### Data Analysis

Our collaborative analysis involved three team members (CB, PT, DS) and followed an iterative process grounded in Braun and Clarke’s reflective thematic analysis of familiarisation, identifying, coding, and reviewing and refining themes (Braun & Clarke, 2021). To enhance our analysis with critical realist grounded theory, we integrated a deep emphasis on the underlying mechanisms that shape participants’ experiences. Building on Braun and Clarke’s reflective thematic analysis, we not only followed the iterative steps indicated in Braun and Clarke (2021) but also considered the socio-structural factors and individual agency, and ways in which they interacted to influence participants’ experiences and perceptions.

We transcribed focus group discussions verbatim and subsequently uploaded transcripts and free-text comments to NVivo 14 (Lumivero, 2023). We approached coding with a critical realist lens. Our analysis involved not just categorising data into basic codes and sub-themes but also interrogating the social contexts that give rise to the identified patterns. Examples here include behavioural support in dementia, driving restrictions, safeguarding, impact on children and family, and the interface between aged care and disability. Other sub-themes, such as diagnostic experiences, the age-appropriateness of services, work-based training related to young-onset dementia, service accessibility, and available supports, were critically examined to understand structural factors. This analytical approach included consideration for variable healthcare systems, geographical differences, and societal attitudes toward young-onset dementia influencing participants’ subjective experiences.

During our coding sessions, we undertook collective team discussions that not only focused on the accuracy of codes but also explored the interplay of individual experiences and broader social forces and expectations. Our collective inquiry aimed to address discrepancies in interpretation by considering the impact of power dynamics, social norms, and institutional frameworks on the participants’ experiences. This collaborative evaluation ensured that all team members contributed to defining and refining the thematic framework.

We synthesised sub-themes into major themes to enhance emergent patterns visually. We reflected on the complex interactions between individual experiences and structural conditions. This approach allowed us to dynamically explore relationships between sub-themes and major themes within specific social and structural contexts, highlighting the mechanisms that influence participant experiences. This phase of analysis was supported by real-time discourse, emphasised patterns, and the generation of themes that captured both individual voices and nuanced realities. By incorporating critical realist grounded theory into our framework, we aimed for a rich analytical narrative that acknowledges the interplay of agency and structure in shaping experiences relating to young-onset dementia.

The iterative nature of our thematic analysis and the process used for collaborative decision-making produced clustered themes. We then secured an overarching descriptive for each cluster. For example, content relating to public awareness of young-onset dementia, primary health care understanding, specialist knowledge, workforce training, information resources, and knowledge within non-dementia service settings were themed into ‘*Knowledge, Information and Education’*.

Finally, we used numerical identifiers or quotes provided by focus group participants. However, quotes provided by free-text comments were indicated as such.

## Findings

We analysed qualitative data from ten focus groups and optional free text comments, where provided, from 313 completed surveys. Demographics are presented in Table 1.

**Figure 1.**
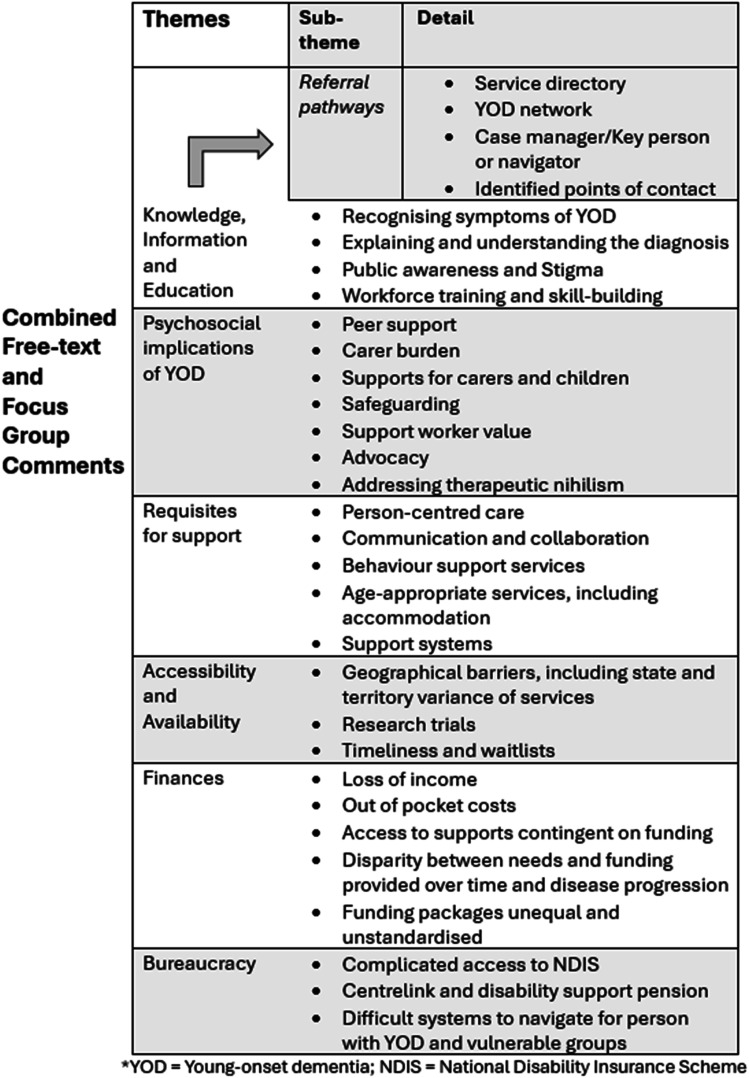
Six Qualitative Themes

Our results identified six high-level themes from the *Joint Solution*s project qualitative data. Major themes are displayed in Figure 1.

### Themes Identified from the Analysis

#### Knowledge: Information and Education

##### Workforce Training and Skills Building

Our analysis identified significant gaps in the specialised knowledge of young-onset dementia along key aspects of the care pathway. Participants emphasised the necessity for a well-informed workforce, including knowledgeable general practitioners (GPs) and service providers. Participants noted that young-onset dementia extends beyond memory deficits, which they felt is not well recognised in the current care system. Furthermore, participants identified a pressing need for targeted public education to enhance awareness of young-onset dementia and mitigate stigma, alongside more structured training for professionals working within health and disability services.

“I’ve been giving so many education and information sessions to support coordinators because they actually do not understand dementia, so they don’t know how to provide the services” (Community Service Provider #1)

“There’s an expectation that there’s going to be a level of knowledge and understanding around younger onset dementia… and from my experience, often, be it a support worker or a GP, there isn’t that level of knowledge or understanding” (Community Service Provider #9)

When seeking initial consultations with their GPs after experiencing symptoms, participants reported a variety of experiences. Some expressed concerns about their GPs’ limited understanding of young-onset dementia and felt that their symptoms were often misattributed to more commonly recognised psychiatric conditions. This left them feeling misunderstood and frustrated. In contrast, others shared positive experiences, highlighting GPs who were knowledgeable and responsive, initiating timely and appropriate care.

“I went to the GP and I told him I have frontotemporal dementia. And he said, ‘Oh, no, you’re just busy with your life, children and your old parents, and you're just exhausted... You’re too young to have a dementia.’ So, this GP certainly didn’t know anything about young-onset dementia” (Person with young-onset dementia #3)

“Our GP was very well informed. He had been looking after [her] and had diagnosed with generalised anxiety disorder, so when we noticed a change, he took that seriously” (Caregiver #12)

Some participants with lived experience shared that after receiving their diagnosis, they needed more information about young-onset dementia in general, and their specific type of young-onset dementia, than what was initially provided. They emphasised that the diagnosis and accompanying information should be delivered with greater empathy. However, others expressed that they needed time to absorb the diagnosis. They felt overwhelmed when it was first communicated and struggled to process initial information, but did require ongoing information during subsequent appointments.

“My neurologist spoke the diagnosis into his dictaphone and ushered us out to pay” (Person with young-onset dementia free-text)

“My diagnosis was explained as fatal, with a life expectancy of 7-9 years, which has not been my experience. Almost no other information was provided, and no referrals to support” (Person with young-onset dementia free-text)

“So, when she said… I don’t really need to see you again, and I thought, well, what do I do? Where do I go for information?” (Person with young-onset dementia #2)

Once diagnosed, additional concerns were raised about the systemic nature of knowledge deficits related to young-onset dementia across support services. Participants highlighted the National Disability Insurance Scheme as a key service provider and emphasised the need for a better understanding of the heterogeneous characteristics of young-onset dementia to inform its service provision. This included more effective responses to people experiencing changed behaviours.

“Even though we have good [National Disability Insurance Scheme] plans for them, we often see that disability support organisations don’t have the training to be able to adequately support people with a younger onset… I find that we have funding in a plan, but we simply can’t utilise it because we don't have the trained support staff” (Community Service Provider #6)

#### Referrals

Participants noted the lack of referrals to allied health professionals, such as psychology, family counselling, genetic testing, and speech pathology, which were deemed essential but underutilised after the diagnosis of young-onset dementia. Some clinicians mentioned specific barriers to providing these referrals, such as eligibility criteria, costs, and uncertainty about whom to refer to or where to send clients. When referrals were made, people with young-onset dementia and their families appreciated the access.

“…Public sector referral to psychiatrists is actually extremely difficult, and quite a few times we’ll refer somebody and be told that, ‘No, they don’t meet criteria, they aren’t going to be seen by neuropsychology.’” (Clinician #2)

“We were referred to a [Dementia Behaviour Management Advisory Service] person who was wonderful. And that was done by the second neurologist who asked us if we’d like to have that connection” (Caregiver #12)

Participants expressed a desire for a more streamlined and consistent referral system. Some suggested that a resource could be developed to guide and standardise care, including referrals.

“A clear pathway… That the person that makes that diagnosis knows very clearly this is the pathway you need to go on. This is where I'm referring you to… a clear pathway” (Caregiver #10)

“[There is a] lack of knowledge of where to refer people to for investigation of cognitive changes” (Community Service Provider free-text)

Participants with lived experience wanted a case manager or key navigator person to help connect them to a multidisciplinary team and access necessary services. Some recalled the previous young-onset dementia key worker program and suggested reinstating this role to facilitate access to relevant information and support, and to navigate the care system.

“But having a key worker meant that we felt like there was always someone we could go to with questions” (Caregiver free-text)

“I really think having somebody to help you from the word go would really be great” (Person with young-onset dementia #5).

“The [young-onset dementia] key worker model worked well as they walked the journey with the person and their networks, providing information and support, opting in and out as the stages progress” (Clinician free-text)

Moreover, participants felt that formal support should include assistance with workplace challenges, financial planning guidance, and more provider recognition of remaining active in the community. However, it was unclear whether participants believed these supports should be provided within the structure of the National Disability Insurance Scheme.

#### Psychosocial Implications

Participants emphasised the need for services that address the psychosocial implications and consequences the diagnosis has for employment status, self-identity, and purpose. They felt psychosocial support was important in the process of acceptance and managing the emotional challenges relating to disease progression. In addition to professional psychology services, some lived experience participants benefited from advocacy roles, peer-to-peer support, and counselling.

“I found with all the advocacy work that I do and that others here do too, it’s really helped keep me active, and I think it’s probably done a bit of the retraining of the brain” (Person with young-onset dementia #5)

“The advocacy system is good because it gives [some people] a focus and gives them a community that they can deal with” (Person with young-onset dementia #7)

“Communication with others in a similar situation is probably the most beneficial thing everyone can have. Raises their own expectations, when they can see what other people can still do as opposed to what they’re told they can’t do” (Person with young-onset dementia #1)

Our findings show that participants with lived experience had significant concerns regarding genetically inherited young-onset dementia and its potential impact on children. Clinicians were aware of these concerns but felt it important to introduce the topic carefully and at a time when people are open to considering genetic counselling and testing.

“Often the children are thinking about it, even if it’s not sort of proactively raised… it’s really weighing heavily on their minds often, you know, if their father developed dementia in their 50s, they’re thinking… ‘Should I start a family?’ and all those sorts of things” (Clinician #3)

“I had to push for a genes test with my GP as my dad and his brother both had a dementia. We don’t know if their parents might have had a dementia. I was told by my GP I had a faulty gene for Alzheimer’s” (Person with young-onset dementia free-text)

“...whether to sort of pursue diagnostic genetics is always very, very contingent on the family and particularly considering the wishes of any at-risk children in making those those decisions” (Clinician #3)

Caregivers felt susceptible to high levels of care-related challenges, which they identified as a major cause of psychological distress. However, limited support was available unless personally funded, and even less was available for children with a parent with young-onset dementia. Caregivers generally expressed a desire to access family caregiver programs to help them develop coping strategies, but many felt that more support services are needed to reduce care-related challenges. They suggested family counselling, bereavement support, assistance with future planning, and resources to help keep the family aligned and working together.

“There’s care and support for the people with disability, but there’s nothing for us, as the carers” (Caregiver #2)

“… There aren’t a lot of services available at that family level to maintain that family unit and to support the children and everybody else who’s going through that diagnosis” (Clinician #10)

“Somebody should be checking up on you to see that you are coping because if you’re not coping, then your loved one isn’t” (Caregiver #9)

Support workers were often seen as providing a valuable service for people with young-onset dementia and able to provide some respite for family caregivers. However, support workers expressed a need for work-based training and skills development to better understand how to provide young-onset dementia-specific support and feel better prepared to manage any changed behaviours.

#### Requisites for Support

Several requisites were identified as essential in providing optimal care for people with young-onset dementia and their families. Service providers generally emphasise the importance of person-centred care practices that recognise the unique needs, preferences, values, and circumstances of each person with young-onset dementia.

“The person at the centre of the care has to be the person with the diagnosis of dementia. Not the carer, not the organisation that’s doing it” (Person with young-onset dementia #1)

However, caregivers often felt services lacked person-centred care and life-stage relevance. They provided examples where people were placed in activities or respite care that were inappropriate for their younger age, energy levels, or interests. The lack of age-appropriate services was particularly noted as deficient.

“Clients with [young-onset dementia] have almost no options for day respite, respite or social options that are age-appropriate” (Community Service Provider free-text)

When the needs of a person with young-onset dementia exceed the current support being provided, those funded by the National Disability Insurance Scheme typically require a reassessment or review of their care plan to access a higher level of support. Plan reviews take time to implement, often involving discussions with the agency planner, reassessing current goals, needs, and supports, as well as providing additional documentation necessary to support a plan review request. This process has raised concerns about the National Disability Insurance Scheme’s ability to respond swiftly to rapidly changing care needs, particularly regarding services for managing altered behaviours associated with young-onset dementia.

“More needs to be done in this area [altered behaviours], and the role of communication impairment in behaviour change needs to be acknowledged as well” (Clinician #7)

“Services are not responsive to fast-changing and episodic behavioural and psychological changes” (Caregiver free-text)

“It’s by the time the behaviours are high risk, that’s when suddenly I’m called on. The staff are burnt out, families are burnt out” (Clinician #1)

Effective communication and collaboration systems are essential for providing quality care. One service provider shared an example of successful teamwork, discussing how a productive relationship has developed between their team and a neurologist specialising in young-onset dementia. This collaboration ensured that appropriate, sufficient, and supportive information was readily available for the care of individuals.

“So we have a memory clinic that is part of the Central Health District, but they work well. They’ve got a neurologist on board who’s very [young-onset dementia] -focused… There’s the start of a relationship between that memory clinic and [us] so there’s an automatic feed-through, I suppose… Basically when health network providers are willing to communicate… that’s what works well” (Community Service Provider #9)

However, participants observed that issues relating to communication and collaboration remained prevalent at multiple levels of the care system, including long wait times for some services. They expressed frustrations with inadequate multidisciplinary collaboration and lack of interagency communication. Many people with young-onset dementia were concerned about having to repeatedly share their stories with different service providers, even within a National Disability Insurance Scheme care plan. Participants emphasised the need for agencies to cooperate more effectively, eliminating the need for multiple service assessments, and proposed the creation of an informational system to provide all stakeholders with access to relevant assessments, diagnostic information, and service notes necessary for their roles.

“Nobody knows anything. Nobody’s communicating. There's no collaboration. It’s lots of [siloed] health professionals who are doing their individual assessments, their individual therapy” (Community Service Provider #1)

“[National Disability Insurance Scheme] is a very disconnected system with a lot of wasted money on duplication and lack of communication. This might be improved by creating a shared documentation system, like a hospital setting, where health professionals can read others’ notes/assessments to help create collaboration, connection and person-centred care” (Clinician free-text)

#### Accessibility and Availability

##### Geographical Barriers

People living outside major metropolitan areas often faced challenges accessing young-onset dementia -specific services and programs. Those in regional and rural areas perceived their location as a disadvantage because most diagnostic and post-diagnostic services were generally concentrated in urban areas. This limited access to resources, psychosocial supports and research trials. However, participants generally understood the difficulties involved in providing a comprehensive range of young-onset dementia services outside major urban areas.

“In regional, remote areas, the primary gaps are in the resources to provide both services and education for not just the persons being impacted… All we see in regional areas are organisations closing and funding being cut” (Community Service Provider free-text)

“We have developed a local volunteer [young-onset dementia] service in our area due to the lack of initiatives in our regional area. It is very frustrating…” (Clinician free-text)

In addition, some participants remarked on limited regional transport options, making accessing services outside their immediate locality difficult. They emphasised the importance of online programs being available as an alternative.

Respite and accommodation services were hard to find and, for some, cost prohibitive.

“When [he] had a sharp and unexpected health and behavioural decline, it was very difficult to find respite care - as there are no [National Disability Insurance Scheme] -specific placements in our area and [we are] not eligible for others because [he’s] not on [an] aged care package” (Caregiver #23)

However, a few participants shared positive experiences with respite services, citing well-staffed, welcoming facilities that offered opportunities for social engagement. Though valuable, these examples were scarce and dependent on location and funding availability.

Housing and long-term accommodation were seen as problematic due to scarcity, and those available often lacked adequate support for people with young-onset dementia. There was consensus that young-onset dementia -appropriate housing is a significant area requiring investment.

“Typical disability housing (Supported Disability Accommodation and Supported Independent Living) does not meet the needs of people with [young-onset dementia] due to lack of care staff experience in supporting people with these conditions” (Clinician free-text)

“My wife doesn't fit into a residential model that can be supported in our area. She is 63.5 years old and all aged homes in our area refuse to accept her” (Caregiver free-text).

“This is a long and challenging process to navigate, and finding suitable accommodation for [young-onset dementia] for a person's individual needs is very difficult. The available [options] are usually set up for developmental disability clients or psychiatric clients, and thus, the unit managers and their support worker team are NOT adequately informed or skilled to deal with the often complex and evolving challenges experienced by [young-onset dementia], especially those with behavioural/frontal lobe issues” (Clinician free-text)

#### Finances

Many participants with lived experience acknowledged financial hardship resulting from loss of income and altered circumstances. In addition, out-of-pocket costs were reported for both diagnostic and post-diagnostic tests and services, including functional capacity assessments needed for National Disability Insurance Scheme access. Participants noted that obtaining post-diagnostic services was often contingent on receiving funding, such as through the National Disability Insurance Scheme. Without this funding, many services did not accept referrals, and even when funding was in place, some participants still incurred hefty out-of-pocket costs to supplemental services.

“People are not, you know, sometimes able to pay … up to $400.00 for a functional capacity assessment” (Community Service Provider #4)

“It was costly and time-consuming to get re-evaluations and reports ready, and then at the review, some of these were clearly not read” (Caregiver free-text)

Most participants with lived experience were supported by the National Disability Insurance Scheme, but those who were aged 65 years and older accessed less comprehensive aged care packages, leading to disparities in services and funding. Some participants noted that National Disability Insurance Scheme funding was inconsistent, even among those with similar needs. As the disease progressed, discrepancies emerged between recipients’ needs and the funding they received, often necessitating costly plan reviews. At the systems level, clinicians and service providers highlighted inadequate funding for clinical and community services and programs, which limited access to essential resources, reflecting broader systemic shortages.

#### Bureaucracy

Excessive bureaucracy was seen as a significant and unnecessary source of distress. Applications for governmental benefits (e.g. Centrelink) and disability support pensions were described as burdensome, and applying for National Disability Insurance Scheme access was reported to be challenging due to lengthy, complex forms and subsequent plan reviews. The onus of completing these forms often fell on the person with young-onset dementia or their caregiver.

“My husband has been eligible for a part disability support pension and myself for carer’s payment for over 12 months, but the process is long and demanding. I have commenced applications a number of times and just given up because I don’t have the time or energy to deal with another stressful task” (Caregiver free-text)

“[National Disability Insurance Scheme] plans are so hard to understand; they use weird descriptions of what each pot of money is for. Centrelink demanding more medical forms to be filled in for carers payment when multiple tests have already been done for [National Disability Insurance Scheme]” (Caregiver free-text)

“We’ve had enduring power of attorney done for a good 20 years, and sometimes it’s just worth nothing, like in the banks” (Caregiver #3)

**Figure 2.**
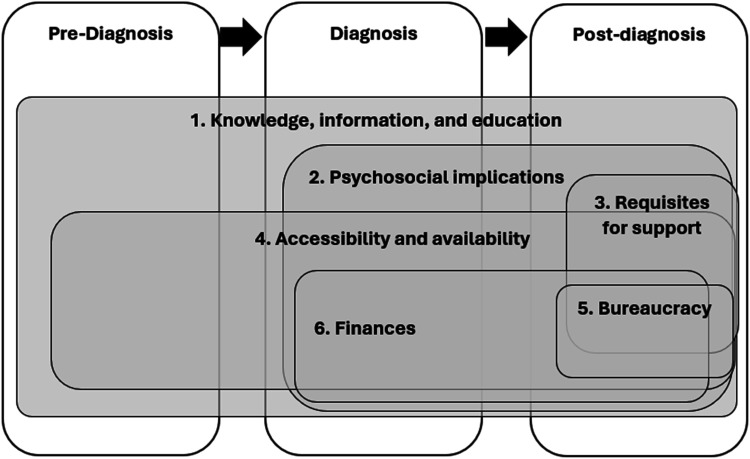
Themes Summary Graphic

Figure 2 summarises the themes and illustrates their position on the young-onset dementia care pathway and areas of overlap.

## Discussion

Our findings highlight the complex nature of young-onset dementia, and the significant adjustments required by individuals and their families (Aspö et al., 2024). The implications for work, relationships, finances, and psychosocial needs are profound (Draper & Withall, 2016). How services interact with each other and communicate with individuals and their families is paramount, as it shapes both diagnostic and post-diagnostic experiences. However, we found that service provision continues to vary across Australia due to its size, rurality and demographics. This makes consistent and equitable access to young-onset dementia services challenging, reflecting systemic issues leading to inequitable care. While these challenges may seem insurmountable, solutions do exist. The findings of the *Joint Solutions* project recommend overcoming geographical barriers through a hub-and-spoke model of care (Loi et al., 2024), providing multidisciplinary diagnostic teams and improving access in regional, rural and remote communities (Burkinshaw et al., 2023). Web-based resources can be centralised to create an information hub (Loi et al., 2024). Expansion of telehealth can build on the positive experiences of clinicians already using this method to connect with people with young-onset dementia (Brown et al., 2023). These are all promising strategies to equalise access to young-onset dementia services across Australia.

Participants in this study highlighted the significant impact that young-onset dementia has on both individuals and their families. Receiving a diagnosis is devastating and carries far-reaching consequences (Draper & Withall, 2016; O’Malley et al., 2021). Yet the diagnostic process remains susceptible to the same findings identified in an earlier study, including low awareness of young-onset dementia within the community, a lack of recognition of cognitive changes, and frequent early misdiagnosis as a mental health condition (Draper et al., 2016). Moreover, applying to the National Disability Insurance Scheme for post-diagnostic services is bureaucratic and complex (Carey et al., 2021). Simplifying and streamlining application processes based on the receipt of a diagnosis would significantly improve the care experience. However, National Disability Insurance Scheme eligibility is determined by current need rather than future ones. Access to more sophisticated diagnostic techniques, such as early symptomatic biomarkers can lead to earlier diagnosis, which may create challenges in meeting National Disability Insurance Scheme eligibility if impairment is mild (Cations et al., 2022). Additionally, individuals still express concerns about the ability of the National Disability Insurance Scheme to provide them with necessary services. These concerns include navigating the system and identifying appropriate supports for specific needs, especially when considering the progressive nature of young-onset dementia. Such issues can complicate the process of utilising allocated funding and achieving well-integrated care. Despite these difficulties, the overall perception of the National Disability Insurance Scheme remains positive, particularly due to its focus on disability and rehabilitation to promote social participation, rather than being embedded in aged care (Cations et al., 2022).

Care-related challenges frequently precipitate significant psychological distress (Kang et al., 2022; Lim et al., 2017). However, access to psychology-based resources, which are essential for managing emotions and coping with the progression of symptoms, remains markedly inadequate (Grunberg et al., 2022). The broader integration of psychological services within the Australian healthcare system, alongside the establishment of a structured referral pathway at the point of diagnosis could enhance accessibility, particularly through the utilisation of telehealth modalities. Furthermore, the financial strain associated with care, which is intensified by increased caregiver needs, loss of employment, and out-of-pocket expenditures, exacerbates the adverse ramifications of young-onset dementia and disproportionately affects lower socio-economic groups (Kilty et al., 2023).

Participants identified the need for improved support to navigate systems. Several recommended that a revived, sustainable version of the previous young-onset dementia key worker program is needed to work alongside individuals and their families to provide specialist knowledge, advice, support navigation, improve caregiver self-efficacy, and promote equitable access to services such as the Care Ecosystem model (Guterman et al., 2023). This may be particularly useful for socio-economically disadvantaged groups, culturally and linguistically diverse and First Nations communities struggling to access post-diagnostic and National Disability Insurance Scheme support (Carey et al., 2021; Cortese et al., 2021).

Previous research indicates that mainstream dementia services were predominantly designed to meet the needs of people with late-onset dementia (Bakker & Appelhof, 2021). Our study raises concern that, despite previous identifiers and calls for improved young-onset dementia services (Cations et al., 2021; Ottoboni et al., 2021; Stamou et al., 2022), these services remain lacking. People with young-onset dementia are often more aware of the disease’s impact on their role and status than their older counterparts, making their care needs distinct (Bakker & Appelhof, 2021; Baptista et al., 2019; Mayrhofer et al., 2018). This underscores the need for a young-onset dementia-informed, person-centred, and collaborative care pathway, where key roles and responsibilities are clearly defined across diagnostic and post-diagnostic stages (Cations et al., 2021; Loi et al., 2024). Similar needs have been identified internationally (Mulhall et al., 2024; Stamou et al., 2022). Whilst our findings align with other studies, they also highlight the unique challenges of the Australian care system and its geographical complexities.

Our study highlights a concerning lack of young-onset dementia understanding across professional services, which is essential for effective care delivery (Couzner et al., 2022). To address this, young-onset dementia-focused professional development programs should be widely implemented to ensure services align with best practice (Annear, 2020). Additionally, greater uptake of resources such as the Massive Open Online Course (MOOC), which has been shown to improve dementia knowledge in both professional and family caregiver settings, should be encouraged (Eccleston et al., 2019). Increasing public awareness of young-onset dementia can also promote early symptom recognition, encourage GP consultations, and address a critical knowledge gap of dementia and its risk factors. Improved understanding of dementia may be particularly relevant for young adults who have the most potential to mitigate their dementia risk (Keage et al., 2021).

This study, developed from empirical evidence, contributes to the discourse on stakeholder expectations and requirements in young-onset dementia care. A key strength of this paper is its inclusion of perspectives from people with YOD, their caregivers, clinicians, and service providers. However, participants were predominantly Caucasian, lived or worked in metropolitan areas, and were educated. We had limited representation from First Nations peoples, lesbian, gay, bisexual, transgender, intersex and other diverse sexualities, or from other minority groups, as well as under-representation from stakeholders in the Northern Territory.

To address the limitations identified in this study, future research should adopt more inclusive and targeted recruitment strategies to ensure broader representation across cultural, geographic, and socioeconomic groups. Specifically, greater efforts are needed to engage with minority communities by partnering with trusted community-based organisations and employing culturally responsive research methods. For example, working more directly with Aboriginal Community Controlled Health Organisations and cultural services specific to minority communities. Expanding recruitment beyond metropolitan areas, particularly to include regions like the Northern Territory, is essential for capturing data from diverse communities, including greater utilisation of region-specific outreach services. Telehealth tools, and alternative data collection methods can enhance participation from rural and remote stakeholders. Incorporating diverse language-specific methods for data capture should facilitate greater representation from minority communities.

In addition, future research could corroborate with studies already undertaken that include data on diagnosis, care and management of minority communities in Australia. Australian-based studies in young-onset dementia are limited. However, Burkinshaw et al. (2023) provide insight into system and policy barriers in young-onset dementia in First Nations people, other culturally and linguistically diverse people, and those living in rural and remote Australia. Cations et al. (2021) highlight actions to improve identification, diagnosis, treatment and care in geographically dispersed people with young-onset dementia, along with improved diagnosis and management for Australian First Nations people. Li et al. (2014) found an increased prevalence of young-onset dementia among Australian First Nations people in Australia’s Northern Territory.

## Conclusion

While Australia has made progress in supporting people with young-onset dementia, diagnostic and post-diagnostic care remain nationally inconsistent and need improvement. Implementing a strategy for a nationally delivered, evidence-based, and streamlined young-onset dementia specialist service is crucial. Care must be flexible and collaborative, supported by a knowledgeable workforce, and led by clear guidelines. Within this model, priority areas include having improved resources for young-onset dementia, addressing the age-appropriateness of services, expanding access to psychosocial support, and ensuring sufficient respite and accommodation options exist for people with young-onset dementia.

## Data Availability

Participant data accessed can be discussed with Dr. Clare Beard.*
